# Setting the record straight: Loss of wall-associated kinases does not affect plant perception of pectin fragments

**DOI:** 10.1093/plcell/koae318

**Published:** 2024-12-09

**Authors:** Leonard Blaschek

**Affiliations:** Assistant Features Editor, The Plant Cell, American Society of Plant Biologists; Department of Plant & Environmental Sciences, University of Copenhagen, Thorvaldsensvej 40, 1871 Frederiksberg C, Denmark

The story of the Wall-Associated Kinases (WAKs) began almost 30 years ago. Arabidopsis (*A. thaliana*) WAK1 was identified in plasma membrane fractions strongly bound to cell walls, suggesting a role in maintaining the interface between the 2 compartments (He et al. 1996). Early attempts at reverse genetics, antisense constructs targeting all 5 Arabidopsis *WAKs*, yielded no viable seeds and the assumption that a complete loss of the encoded proteins was lethal (Wagner and Kohorn 2001). Over time, gain-of-function evidence accumulated that suggested WAKs could perceive oligogalaturonides (OGs), fragments of cell wall pectin produced during biotic attacks. This role was seemingly solidified by independent *WAK* mutants published in recent years. The allele published by Kohorn et al. (2021), *wakΔ*, still contained a chimeric *WAK4–WAK2* remnant and showed reduced reactive oxygen species production in response to OGs and the pathogen-associated molecules chitin and flg22. Congruently, the *wakq-1* and *wakq-2* alleles lost the transcriptional upregulation of *Pathogenesis-Related 1* and *2* (*PR1/2*), marker genes of salicylic acid–induced defenses, in response to OG treatment (Liu et al. 2023).

Now, however, the picture has shifted. In new work, **Laura Herold and colleagues (**Herold et al. 2024**)** show that, despite the prior observations to the contrary, WAKs are entirely dispensable for the plant response to OGs. The authors excised the remaining *WAK4–WAK2* chimera from *wakΔ* and tested whether the resulting mutant, called *wakΔ2*, could perceive OGs. Perplexingly, none of the many tested responses to OGs were impaired. In the short term, production of reactive oxygen species, MAP-kinase phosphorylation, and ethylene production were unaffected. Longer-term responses, including callose deposition, growth inhibition, and PR1 protein accumulation in response to OG treatmen, also remained indistinguishable from the wild type. Accordingly, resistance against the fungal necrotroph *Botrytis cinerea* (estimated by lesion size) and bacterial hemibiotroph *Pseudomonas syringae* (estimated by pathogen load) was unaffected in *wakΔ2* plants, with or without prior priming with OGs. Lastly, the authors showed that plant responses to flg22 were equally unchanged. The consistent null results across a variety of covered responses and growth stages—different experiments used plants between 2 and 6 weeks old—compellingly demonstrated that WAKs are not required for OG perception or response.

How then do we explain the discrepancy between these results and the evidence accumulated by the field in the decades prior? And why do 3 loss-of-function alleles seemingly behave so differently? The latter question was raised and addressed during peer review (report available in the Supplementary Data). In additional experiments performed by Herold et al., neither *wakq-1* (Liu et al. 2023) nor *wakΔ* (Kohorn et al. 2021) showed any defects in OG response. Hence, the distinct conclusions were not due to the use of different genetic material (Table 1). The discrepancy with Kohorn et al. (2021) could be due to different growth conditions. The comparison with Liu et al. (2023), on the other hand, offers another reminder that transcriptional upregulation, in this case of *PR1* and *2*, does not guarantee a change in the protein levels.

**Table 1.** Published phenotypes of *WAK* quintuple loss-of-function mutants.

OG response	*wakΔ* (Kohorn et al. 2021)	*wakq* (Liu et al. 2023)	*wakΔ2* (Herold et al. 2024)
ROS production	−^a^	Not tested	0
MAPK activation	Not tested^a^	Not tested^a^	0
Ethylene	Not tested^a^	Not tested	0
Callose	Not tested	Not tested	0
Growth	Not tested^a^	Not tested	0
PR1			
Transcript	Not tested	−	Not tested
Protein	Not tested^a^	Not tested^a^	0
Biotic resistance			
*B. cinerea*	Not tested	Not tested	0
*P. syringae*	Not tested^a^	Not tested	0

+, −, and 0 indicate an increase, decrease, and no change relative to the wild type, respectively.

Abbreviations: MAPK, mitogen-activated protein kinase; OG, oligogalacturonides; PR1, PATHOGENESIS-RELATED 1; ROS, reactive oxygen species.

^a^No difference from the wild type in experiments performed by Herold et al. (2024).

From here, 3 models of WAK function seem plausible: 1) WAKs are OG receptors, but their loss is functionally compensated for by redundant WAK-like or analogous receptors—the stark phenotypic consequences of WAK antisense constructs (Wagner and Kohorn 2001) might be due to unintended targeting of WAK-likes, piercing through this genetic redundancy; 2) WAKs perceive molecules of pathogenic origin, as recently evidenced by the requirement of WAK3 for immune responses to a bacterial peptide (Held et al. 2024); and 3) WAKs perceive the physical link between the plasma membrane and cell wall, either directly or through an apoplastic interaction partner analogous to the interaction between FERONIA and Leucine-rich Repeat Extensins (Dünser et al. 2019). Future work will surely bring us closer to an answer, as long as it is supported as robustly as the present work by Herold et al. (2024) dispelled the field's previous assumptions.

## Data Availability

No original data was generated for this article.

## Dive Curated Terms

The following phenotypic, genotypic, and functional terms are of significance to the work described in this paper:

WAK1 Gramene: AT1G21250
WAK1 Araport: AT1G21250

WAK3 Gramene: AT1G21240
WAK3 Araport: AT1G21240

PR1 Gramene: At2g14610
PR1 Araport: At2g14610

WAK2 Gramene: AT1G21270
WAK2 Araport: AT1G21270

WAK4 Gramene: AT1G21210
WAK4 Araport: AT1G21210

WAK5 Gramene: AT1G21230
WAK5 Araport: AT1G21230
